# Cancer-type somatic mutations in saccular cerebral aneurysms

**DOI:** 10.1038/s41431-024-01765-x

**Published:** 2024-12-12

**Authors:** Behnam Rezai Jahromi, Miko Valori, Riikka Tulamo, Suvi Jauhiainen, Henna Ilmonen, Jonas Kantonen, Minna Kaikkonen-Määttä, Aki Laakso, Seppo Ylä-Hertuala, Juha E. Jääskeläinen, Pentti J. Tienari, Mika Niemelä

**Affiliations:** 1 Department of Neurosurgey, Neurocenter, Helsinki University Hospital, Helsinki, Finland; 2 Neurosurgery Research Group, Biomedicum, University of Helsinki, Helsinki, Finland; 3 Translational Immunology Research Program, University of Helsinki, Helsinki, Finland; 4 Department of Biotechnology and Molecular Medicine, A.I. Virtanen Institute, University of Eastern Finland, Kuopio, Finland; 5 Department of Pathology, Medicum, University of Helsinki, Helsinki, Finland; 6 Department of Pathology, HUS Diagnostic Center, Helsinki University Hospital, Helsinki, Finland; 7 Department of Neurosurgery, Kuopio university Hospital, Kuopio, Finland; 8 Department of Neurology, Neurocenter, Helsinki University Hospital, Helsinki, Finland

**Keywords:** Cancer genetics, Cancer epigenetics

## Abstract

Intracranial aneurysms (IAs) are a major cause of subarachnoidal hemorrhage (SAH) which can have a significant morbidity and mortality. The processes underlying the aneurysm development remains unclear. We performed whole exome sequencing of DNA derived from 20 saccular cerebral aneurysms of 20 patients, followed by somatic variant calling. Eleven (55%) of the 20 patients had detectable nonsynonymous somatic mutations and in total, 48 mutations were detected in the aneurysm samples. The mutations were highly enriched in cancer-related genes and 77% were predictably deleterious. A p.Tyr562Asp somatic mutation was detected in the PDGFRB gene; somatic mutations at the same codon have been reported in fusiform cerebral aneurysms. These results widen the concept on the role of somatic mutations in cerebral aneurysms, indicating their possible role in the more common saccular aneurysm, similarly to the rarer fusiform aneurysm.

Intracranial aneurysms (IAs) are pouches of the arterial wall. The aneurysm may rupture, which leads to subarachnoid hemorrhage (SAH) with high mortalitymorbidity. The most significant population level risk factors for IAs rupture are hypertension, smoking, female sex and old age [1]. There exist also several rare mendelian syndromes with an increased risk of aneurysms and their rupture, the most prominent being autosomal dominant polycystic kidney disease [2]. There are two morphologically distinct aneurysm types, the most common saccular formation (95%) and fusiformic (5%) of reported cases.

Activating somatic variants in the platelet-derived growth factor receptor β gene (PDGFRB) were reported in fusiform IAs [3], an aneurysm type where the artery has dilated around its axis. The most common type of cerebral aneurysm is the saccular aneurysm, where a bulge has formed on top of a narrow or wider stem. Saccular IAs occur in 1–3% of the population and cause 80–85% of spontaneous, non-traumatic subarachnoid hemorrhages. PDGFRB is expressed in vascular smooth muscle cells of the artery wall, among other cell types, and has a role in mitogenic and chemoattractant signaling [4]. Somatic mutations have been recently reported also in the saccular type aneurysms in Japanese cohort [5]. Japanese and Finnish patients have had higher SAH rates than other nations- although this has been also declining probably due to decreasing in smoking rate [6, 7].

In order to investigate the development of saccular cerebral aneurysms, we collected aneurysm tissue samples from two cohorts. Cohort-1 consisted of 6 donors and Cohort-2 of 14 donors (Table 1). Cohort-1 was sequenced by both whole-exome sequencing and amplicon sequencing, Cohort-2 by whole exome sequencing only. The aneurysm samples were obtained as by-product of routine treatment surgery and consisted of arterial wall. For control material, we collected 10 samples from similarly located apparently healthy vascular wall tissue of post-mortem donors. The study was approved by the ethics committee and informed consent was obtained from participants.

**Table 1:** Patient characteristics and somatic variants.

Cohort	Sample	Sex	Location	Aneurysm type	Median coverage	N somatic variants	Median allelic fraction	Variants and Allelic Fractions
1	1	male	AcomA	saccular	176x	0	NA	
1	2	female	MCA	saccular (distal)	142x	6	5,6 %	PDGFRB:NM_001355016:Y498D (6.2%)*, LTBP3:NM_001164266:G1078S (3.5%)*, YARS2:NM_001040436:L216M (2.7%)*, ITGAX:NM_000887:F882C (5.0%)*, RPTOR:NM_001163034:K579E (6.7%)*, MPST:NM_001013436:R21Q (14.0%)
1	3	female	MCA	saccular	153x	0	NA	
1	4	female	MCA	saccular	149x	1	8,2 %	PTPN3:NM_001145372:G481S (8.2%)
1	5	male	MCA	saccular	141x	2	4,4 %	TENM2:NM_001080428:T1254I (4.3%)*, NR5A1:NM_004959:S303N (4.4%)*
1	6	female	VA	fusiform/saccular	137x	2	4,4 %	EVC2:NM_001166136:C378Y (3.7%), CARMIL2:NM_001013838:D645E (5.1%)*
2	7	male	ICA/Pcom	saccular	513x	5	3,9 %	LATS1:NM_001350392:H195Y (4.6%), CTSV:NM_001201575:A229E (3.3%), CUX2:NM_001370598:P447Q (3.3%), ACAN:NM_001135:G609A (3.9%), AHCY:NM_000687:I24V (4.6%)
2	8	male	MCA	saccular	819x	16	3,6 %	PLXNA2:NM_025179:C933F (3.6%)*, XIRP2:NM_001199144:F837C (3.1%)*, TRANK1:NM_014831:T1851S (3.4%), ZBTB38:NM_001350100:N731S (2.9%), HRG:NM_000412:H308Y (3.1%), RASSF6:NM_001270392:R166K (4.5%)*, MYBPC1:NM_001254722:M290R (3.0%), WSCD2:NM_014653:P285A (4.6%), ELF1:NM_001145353:G438A (3.5%), CEBPE:NM_001805:A177P (3.0%), KIF7:NM_198525:V447I (5.6%), ZFPM1:NM_153813:E748X (5.7%), MYO1D:NM_001303279:E657G (5.0%), NOL11:NM_015462:T9K (3.6%), MC4R:NM_005912:V213A (3.4%), ZNF449:NM_152695:V18M (6.4%)
2	9	female	MCA	saccular (distal)	775x	7	3,7 %	SI:NM_001041:R157S (3.6%)*, HGF:NM_000601:D171N (3.4%)*, RBM12B:NM_203390:V225I (4.4%), DCAF12:NM_015397:C335F (3.8%), SOHLH2:NM_001282147:S162F (3.7%)*, RPS6KA5:NM_001322238:G185R (4.0%), DDX24:NM_020414:H661Lfs*23 (3.2%)
2	10	female	MCA	giant fusiform/saccular	695x	0	NA	
2	11	female	PICA	fusiform/saccular	586x	0	NA	
2	12	male	MCA	saccular	666x	0	NA	
2	13	male	MCA	saccular	768x	0	NA	
2	14	female	MCA	saccular	659x	0	NA	
2	15	female	MCA	saccular	699x	1	7,7 %	CAMSAP3:NM_020902:R218X (7.7%)
2	16	female	MCA	saccular	649x	0	NA	
2	17	male	PcomA	saccular	746x	0	NA	
2	18	female	AcomA	saccular	822x	2	4,1 %	NCKAP5:NM_207363:G842R (4.3%), MARK3:NM_001128918:S250X (3.9%)
2	19	female	MCA	saccular (distal)	139x	5	3,6 %	BCL9:NM_004326:Q633E (4.2%), TAS2R41:NM_176883:W102X (3.6%), ADAM20:NM_003814:G593S (3.2%), RYR1:NM_000540:S1770W (2.7%)*, DMWD:NM_004943:Q621P (5.6%)*
2	20	female	Pericallosa	saccular	160x	1	9,5 %	FTH1:NM_002032:D16Y (9.5%)

The data processing and bioinformatics of the samples is described in detail in supplementary materials (S1 and Table S1) [8–12]. Eleven (55%) of the 20 patients in the two cohorts had detectable nonsynonymous somatic variants and in total, 48 variants were detected in the aneurysm samples [13]. Details of the samples, location and type of the aneurysms, sequencing coverages and allele fractions of the discovered somatic mutations are shown in Table 1. Detailed information (including chromosomal location and coordinates,) of the detected somatic variants are shown in Supplementary Tables S2-S3.

Cohort-1 exome data, after successful verification using deep amplicon sequencing, showed 11 exonic somatic variants that lead to an amino acid level change. Somatic variants were found in 4 of the 6 aneurysm samples and were present at an allelic fraction between 2.7 and 14% (median 5.0%). The number of mutations ranged between 1 and 6 for each aneurysm sample.

Cohort-2 included 14 aneurysms which were subject to whole exome sequencing without amplicon verification. From this cohort 37 nonsynonymous somatic variants were discovered in 7 of the 14 donors. The number of mutations ranged from 1 to 16 per donor with a median allelic fraction of 3.7% (range 2.7–9.5%), similar in distribution to the amplicon verified Cohort-1. Patient demographics, type and location of the aneurysms, sequencing coverages and variant information are shown in Table 1.

A unique mutational profile was found in each donor as no mutation or mutated gene was discovered more than once. The list of discovered somatic mutations was enriched in cancer-associated genes (*p* = 6.47E-07, IPA top p-value) as all 48 mutations resided in cancer-related genes (IPA “diseases and bio functions” analysis). Another equally enriched category was “Organismal Injury and Abnormalities” (48/48 mutations). Thirty-seven (77%) of the discovered 48 mutations were classified as probably deleterious by either of the two algorithms used (Tables S2-3).

Our analysis found support for the association of the PDGFRB codon Tyr562 somatic mutation reported earlier by Karasozen et al. [3] in fusiform cerebral aneurysms. We found a p.Tyr562Asp change at an allelic fraction of 6.2% in one of the saccular aneurysms in Cohort-1, present in exome data and confirmed with amplicon sequencing. The PDGFRB mutation carrying saccular aneurysm was located at an atypical distal site. The mutation was predictably deleterious and somatic mutations at this codon are also found in the cancer database COSMIC [11], related to thyroid cancer [14–16].

Cerebral aneurysms have been the target of considerable research effort, but the pathogenesis remains far from clear. In this study, we were able to detect 48 somatic nonsynonymous variants in 11 (55%) out of 20 aneurysms from 20 patients, at a median allele fraction of 3.9%. The observation that these variants in our material were overwhelmingly enriched in cancer related genes suggests that they may have a role in disease pathogenesis, as parallels can be drawn between uncontrolled growth in tumors and the massive unstructured proliferation of smooth muscle cells observed in some aneurysms [17]. Theoretically, some of the variants can be immunogenic, which would enhance inflammation and vascular wall remodeling.

It is of note that three of the five aneurysms with more than two somatic variants were located anatomically more distally from the arterial structure of the Circle of Willis. The distal location is less affected by the hemodynamic stress typically associated with aneurysm formation. The accumulation of somatic variants in these areas may lead to wall remodeling and aneurysm formation in an unusually stable hemodynamic environment. Aneurysms with multiple variants may also undergo both regenerative and degenerative wall remodeling, reflecting the dynamic pathogenesis of the arterial wall. This process might contribute to variant accumulation, as the arterial wall attempts to heal but is drawn back into degenerative cycles. It is clear that some of the variants are “passenger mutations”, particularly in the sample with 16 somatic variants.

Somatic variants have recently been shown to have a credible developmental role in another vascular defect of the brain, namely arteriovenous malformations, in the form of activating KRAS proto-oncogene mutations [18]. Moreover, the recent research performed by Karasozen et al. [3] found four distinct PDGFRB somatic variants in cerebral fusiform aneurysm samples, suggesting a role for somatic mutations in cerebral aneurysm development as well. Among the variants was a p.Tyr562Cys change. Chenbhanich et al. showed that mosaic PDGFRB p.(Tyr562Cys) in ectodermal section of human can cause skin lesions and aneurysm formation [19]. The PDGFRB somatic variant detected in our data was present in the same codon but produced a different substitution, p.Tyr562Asp. The two amino acid changes at this codon are of a different class in terms of physical properties, but both may lead to constitutive activation by interfering with the auto-inhibitory site located within the juxtamembrane region of PDGFRB [20] as predicted by in-silico methods. Recently, somatic variants have been reported also in saccular aneurysms some of which were common in fusiform and saccular aneurysms. The mutations did not enrich to any specific gene in our material. However, in a recent large analysis of 65 IAs from Japanese patients enrichment of variants was found in six genes (PDGFRB, AHNAK, OBSCN, RBM10, CACNA1E, and OR5P3), none of which harbored variants in our analysis [6].

Variants with lower cadd prediction scores are not necessarily more likely to contain cancer-driving mutations, as somatic cancer-driving mutations generally receive higher scores than germline mutations. However, it remains possible that these lower-scoring variants may still contribute slightly to pathogenesis [21].

Further research is needed to analyse somatic variants and their pathways related to cerebral aneurysms. The functionality of these variants potentially driving toward aneurysm formation or vascular healing should be studied in different setup. A more complete understanding of the mutation spectrum may lead to practical applications such as liquid biopsy [22] where circulating mutated DNA can be detected from a patient’s blood sample, and an intervention performed before the rupture of an aneurysm. In addition, pharmacological approaches targeting the mutated genes/proteins may become eligible in the future.

## Supplementary information

Supplementary document

Table S1

Table S2

Table S3

## Data Availability

The datasets generated and/or analyzed during the current study are available from the corresponding author on reasonable request.
