# Supplementary material for: Cancer-type somatic mutations in saccular cerebral aneurysms
Source: Eur J Hum Genet. 2024 Dec 12;33(8):1076–9. doi: 10.1038/s41431-024-01765-x (PMC12322075; doi:10.1038/s41431-024-01765-x)
Supplement: Supplementary file 1 — Supplementary document [file 41431_2024_1765_MOESM1_ESM.docx]

Data processing and bioinformatics of the samples.

DNA was extracted and delivered for whole exome sequencing at Blueprint Genetics (Helsinki, Finland). For sequencing library preparation, the extracted DNA was acoustically sheared (Covaris, City, Country) and sequencing adapters were added by ligation. Exome capture was performed with the SureSelect Human All Exome V6 kit (Agilent, City, Country) and the HiSeq 4000 and NovaSeq 6000 instruments (Illumina, City, Country) were used for next generation sequencing. Data generated by next generation sequencing produced a median coverage of 145x over the exome target regions for Cohort-1 aneurysm samples (range 137x-176x) and a median coverage of 680x for Cohort-2 samples (range 139x-822x).

The somatic variant calling was carried out using a purpose-built bioinformatics pipeline^8,9^ where each of the whole exome sequenced aneurysm samples was called separately, while the control samples acted as a pooled technical reference.

In the bioinformatic pipeline, sequencing reads were mapped to the hg19 reference genome using BWA MEM^9^ followed by duplicate marking with Picard MarkDuplicates (http://broadinstitute.github.io/picard). Adapter sequences were removed before mapping. Somatic variants were called using TNScope^11^ and annotated with ANNOVAR^12^. For the variant calling process, a pooled control reference bam file was created by combining data from all ten unaffected samples. Each affected sample was then called separately with TNScope, paired with the pooled control bam file. A pooled control was used to filter out artefacts caused by common site-specific sequencing and mapping errors. Variant calls from TNScope were then additionally filtered favoring specificity over sensitivity, by first excluding all variants not marked “PASS” by TNScope and then applying a second set of stringent filters (Table S1), in order to remove false positive variant calls and germline variants. Enrichment of genes that contained filter-passing nonsynonymous somatic variants to biological processes was evaluated with the software IPA^10^ (QIAGEN Inc.). Variant deleteriousness was assessed using the PolyPhen2^11^ and CADD^12^ algorithms. To confirm the somatic mutations for Cohort-1, we prepared PCR amplicons targeting each of the variants. The amplicon preparation followed the Illumina 16S protocol with PCR primers designed to target each somatic variant that was called in whole exome data (supplementary material). Amplicons were produced in pairs from the DNA of the sample whose exome data contained the somatic variant, and from a well characterized control NA12878 DNA sample (Coriell Biorepository). Sequencing was performed with the MiSeq instrument (Illumina Inc.). MiSeq sequenced PCR amplicon reads were mapped to the hg19 reference genome using BWA MEM. Samtools mpileup^12^ was used to count the number of read pairs that supported the whole exome somatic variant calls with high quality base calls (Q30+) at the corresponding loci in amplicon data.
