# Supplementary material for: Cancer-type somatic mutations in saccular cerebral aneurysms
Source: Eur J Hum Genet. 2024 Dec 12;33(8):1076–9. doi: 10.1038/s41431-024-01765-x (PMC12322075; doi:10.1038/s41431-024-01765-x)
Supplement: Supplementary file 2 — Table S1 [file 41431_2024_1765_MOESM2_ESM.docx]

| **Default TNScope filters that lead to discarding a somatic variant:** |  |  |
| --- | --- | --- |
| **Filter name** | **Description** |  |
| t_lod_fstar | Somatic variant in the sample does not meet likelihood threshold (the main statistical criterium) |  |
| alt_allele_in_normal | Any evidence of variant seen also in the control bam file |  |
| clustered_events | Clustered events (variants) observed |  |
| homologous_mapping_event | More than three events were observed in the sample bam |  |
| multi_event_alt_allele_in_normal | Multiple events observed the sample and control bam |  |
| str_contraction | Site filtered due to contraction of short tandem repeat region |  |
| triallelic_site | Site filtered because more than two alt alleles pass LOD |  |
| low_t_alt_frac | Site filtered due to low alt allele fraction |  |
|  |  |  |
| **Additional custom filters based on VCF quality metrics used to discard a somatic variant:** |  |  |
| **Variable** | **Failing criteria** | **Description** |
| AF | > 0.2 | Allele frequency, remove germline variants |
| AFDP | < 50 | Minimum sequencing depth to accept variant |
| FS | > 60 | Strand bias, Fisher's exact test score, sites with systematic errors |
| SOR | < 0.25 | Strand bias, odds ratio, sites with systematic errors |
| PV2 | > 0.0001 | Fisher's exact test, control vs sample, similar to t_lod_fstar |
| STR | present | Variant is inside a short tandem repeat |
| GT | not 0/1 | Genotype multiallelic or homozygous |
| AD | < 5 | Variant present in less than 5 distinct supporting reads |
| MQRankSumPS | > 6 | Read mapping quality alt vs ref, Wilcoxon rank sum test z-score |
| ReadPosRankSumPS | < -8 | Variant distance from end of read, Wilcoxon rank sum test z-score |
| ReadPosEndDistPS | < 10 | Mean distance of variant from either end of read |
| ALT_F1R2 | < 1 | Supporting read pairs in F1R2 orientation |
| ALT_F2R1 | < 1 | Supporting read pairs in F2R1 orientation |
| FOXOG | > 0.8 | Fraction of reads with possible OxoG error signature |
|  |  |  |
| **Filters based on ANNOVAR annotations used to discard or include a somatic variant:** |  |  |
| Variant is required to be inside a coding exon |  |  |
| Variant is required to cause an amino acid level change using both RefSeq and GENCODE annotations |  |  |
| Variant may not be present in the ExAC database |  |  |
| Variant may not be present in the gnomAD database |  |  |
| Variant may not be located inside a known segmental duplication region (UCSC Genome Browser table genomicSuperDups) |  |  |
